# Multidisciplinary clinical approach by sharing oral examination information to treat a diabetes patient with dysgeusia

**DOI:** 10.1002/ccr3.2111

**Published:** 2019-03-22

**Authors:** Kazuyuki Matsunaga, Yasuko Yoshida, Makoto Takemaru, Keisuke Yamashiro, Ikuko Monden, Ken Inohara, Saki Nakagawa, Eriko Maeda, Kanako Nakahama, Tatsuo Kohriyama, Shogo Takashiba

**Affiliations:** ^1^ Department of Neurology Brain Attack Center Ota Memorial Hospital Fukuyama, Hiroshima Japan; ^2^ Department of Pathophysiology‐Periodontal Science Okayama University Graduate School of Medicine, Dentistry, and Pharmaceutical Sciences Okayama Japan; ^3^ Keishu‐kai Inohara Dental and Rehabilitation Clinic Fukuyama, Hiroshima Japan

**Keywords:** candidiasis, diabetes, dysgeusia, median rhomboid glossitis, multidisciplinary clinical approach

## Abstract

Taste alteration is one of the complications of severe diabetes. It is important in diabetes treatment to assess taste alteration and perform dietary counseling, therapeutic exercise, and oral care. In this case, multidisciplinary clinical approach by medical staff was successful for a severely diabetic patient with dysgeusia.

## 1 INTRODUCTION

The association between diabetes and dysgeusia has not been understood well. In this case, multidisciplinary clinical approach by medical and dental staff by sharing oral examination information of a severe diabetes patient with dysgeusia improved diabetes, candidiasis, median rhomboid glossitis, and dysgeusia.

Diabetes is one of the lifestyle‐related diseases, and once it becomes severe, it causes various complications, such as retinopathy, nephropathy, and neuropathy.^1^ It is also reported that diabetes induces oral infection such as periodontitis by microangiopathy and macrophage migration inhibition.^2^ Dysgeusia has several symptoms such as impaired or unpleasant taste, and there are various causes. The most common causes could be oral infections, upper respiratory tract infections, and sinus infections.^3^ Diabetes is one of the uncommon causes of dysgeusia, and diabetes itself contains various systemic complications, so it is difficult to clarify the direct relation between both diseases. In this report, we describe a severe diabetes patient with dysgeusia and how multidisciplinary clinical staff such as physicians, nurse, nutritionist, dentist, and dental hygienist shared her oral examination information, and finally how her general health conditions had improved.

## 2 CASE REPORT

### 2.1 Patient information

A 64‐year‐old Japanese woman was transferred to the hospital due to loss of consciousness. During the anamnesis, she referred a taste disorder whenever she ate that started 10 days before the hospitalization. She had a history of high blood pressure and dizziness but she did not take any medicine for that. She lived with her son and did not visit any hospital for 10 years. Her favorite foods were fruits, pickled vegetables, and coffee. She had an irregular eating pattern.

### 2.2 Physical findings at admission

On the admission day, the patient's temperature was 31.4°C, her blood pressure was 94/60 mm Hg, and her pulse was 73 beats/min. She was 151 cm tall in height, 76.8 kg in weight, and the BMI at that time was 33.7 kg/m^2^. Her blood test results showed high regarding fasting blood sugar (1348 mg/dL), HbA1c (15.8%), urea nitrogen (100.5 mg/dL), creatinine (3.94 mg/dL), and low levels of serum iron (21.0 μg/dL). We diagnosed her as type 2 diabetes with hyperosmolar hyperglycemic syndrome and dysgeusia. She received treatment with rehydration and insulin injection. Due to anorexia and dysgeusia, she was referred to the department of dentistry in the hospital 4 days after hospitalization.

### 2.3 Intraoral finding at dental examination

**Figure 1:**
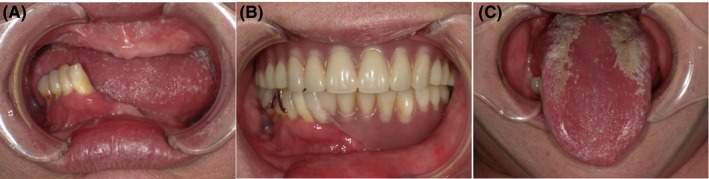
Intraoral findings at dental examination (A) only four teeth left in the right lower mandible, (B) upper and lower well‐fitting dentures, (C) red and smooth tongue, white lesion on both side

There were only four teeth left in the right lower mandible, and she was using upper and lower well‐fitting dentures (Figure 1A,B). Her oral mucosa was dry and red. Her tongue was red and smooth in the middle, and there were white lesions on both sides (Figure 1C). We detected *Candida albicans* from the white lesions by bacterial examination. Salt‐impregnated test results showed lower sensitivity to salty taste. An additional blood test results showed low serum zinc (52.0 μg/dL; lower limit: 57‐65 μg/dL). We diagnosed her as candidiasis, median rhomboid glossitis, as the main reasons for her dysgeusia.

### 2.4 Treatment progress

**Figure 2:**
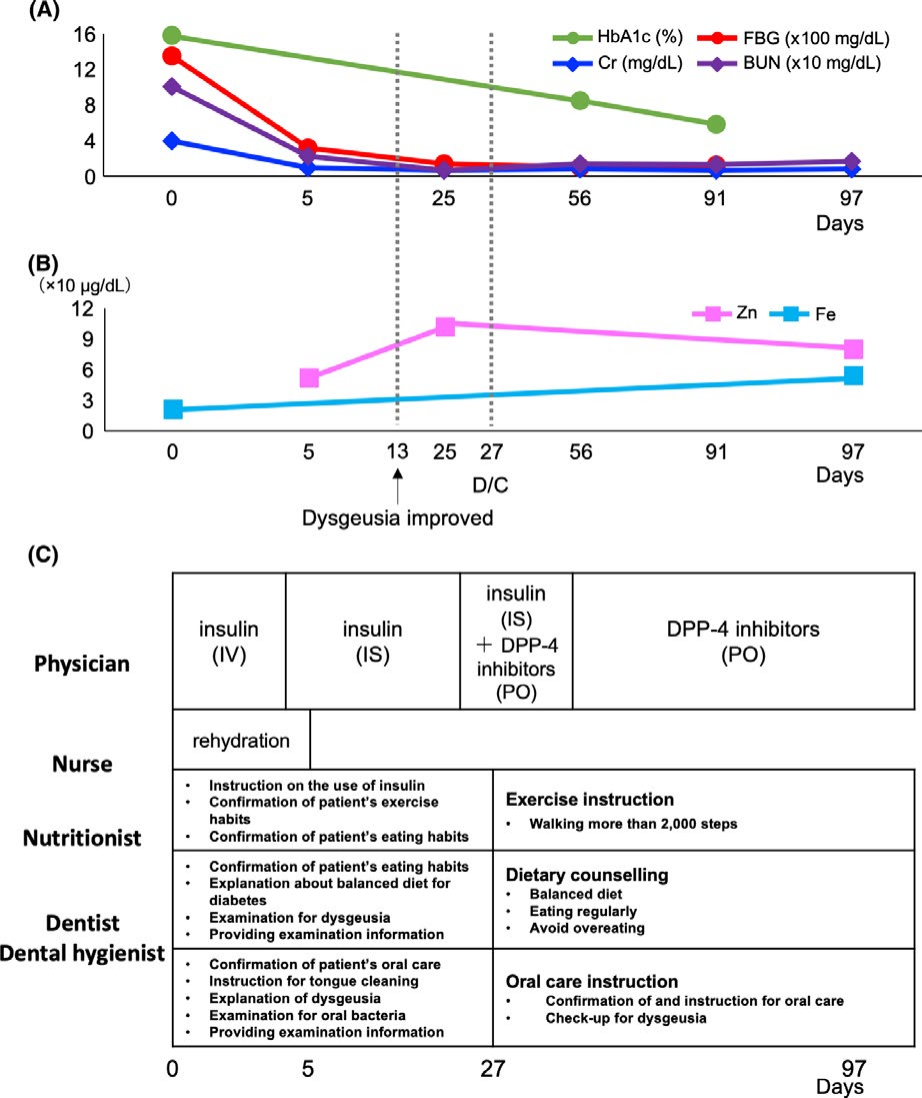
Treatment progress. A, The changes in HbA1c (green line), FBG (fasting blood glucose; red line), Cr (creatinine; blue line), and BUN (blood urea nitrogen; purple line); B, The changes in Zu (serum zinc; pink line), and Fe (serum iron; sky blue line); C, Multidisciplinary team's treatment plan

**Figure 3:**
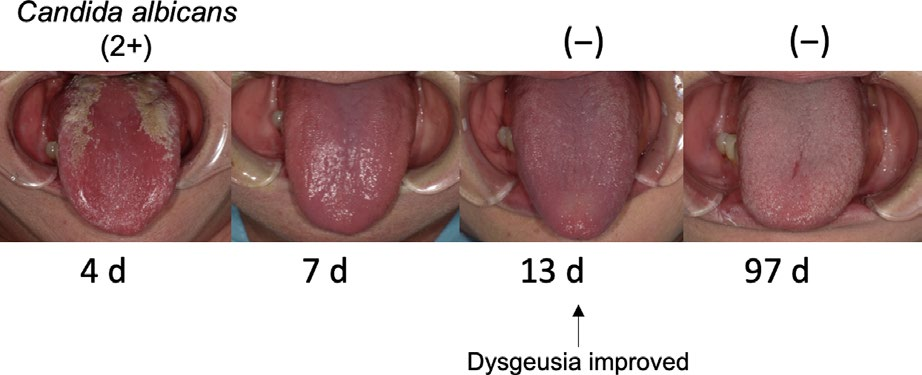
Post‐treatment tongue's improvement and decrease in *Candida albicans*

By rehydration and insulin injection, her blood sugar and renal function were improved 5 days after hospitalization (Figure 2A). Nurses taught her as therapeutic exercise to take at least 2000 steps in a day (Figure 2C). Nutritionist taught her as dietary counseling to take well‐balanced diet, such as seafood contained zinc, and avoid overeating (Figure 2C). In dentistry, dentist and dental hygienists performed oral care, especially tongue cleaning, and bacteria examination routinely (Figure 2C). The oral conditions and bacterial examination results were shared with the multidisciplinary team. Thirteen days after hospitalization, *C. albicans*fell below measurable limits, and her median rhomboid glossitis and dysgeusia were improved (Figure 3). Twenty‐seven days after hospitalization, she was discharged from hospital since her general health condition had been recovered. Ninety‐seven days after hospitalization, she visited the hospital due to the check‐up of her condition. Her weight was 62.7 kg (−14.1 kg), and BMI decreased 27.7 kg/m^2^ (−6.2 kg/m^2^). Her blood test results also showed improvement regarding fasting blood sugar (121 mg/dL), HbA1c (5.8%), urea nitrogen (16.5 mg/dL), creatinine (0.77 mg/dL), serum iron (54.0 μg/dL), and serum zinc (80.0 μg/dL) (Figure 2A,B). She got used to have regular eating patterns, to eat seafood, and avoid to eat a big quantity of fruits. Now she takes 5000‐7000 steps in a day.

## 3 DISCUSSION

Dysgeusia has been defined as a disgusting taste or altered taste sensation. There are three different varieties of dysgeusia: hypogeusia is defined as a reduction in one of four, or all four taste modalities, such as sweet, salty, sour, and bitter. Ageusia is defined as a complete lack of taste. And finally, allotriogeusia is defined as an irregular perception of taste.^4^ The causes for these taste‐related disorders could be as follows: structural change of tongue surface such as lingual papillae by anemia, decreased salivary secretion by aging or Sjögren's syndrome, candidiasis, radiation therapy, zinc or copper deficiency, medicine intake (antihypertensive, tranquilizer, antibiotics, anti‐allergic), periodontitis, and so on.^5^ In this clinical case, we considered complex factors that could cause the dysgeusia. The patient had severe diabetes, and it might induce iron‐deficiency anemia, zinc deficiency, and candidiasis. Furthermore, diabetes also induced hyperosmolar hyperglycemic syndrome caused by dehydration, following iron and zinc deficiency. Diabetes‐induced compromised condition also caused candidiasis following median rhomboid glossitis. From these results, we concluded that she had dysgeusia (it might be allotriogeusia because she felt a bitter taste whenever she ate).

Bajaj et al reported that in 50 cases of type 2 diabetes mellitus (DM), oral manifestations included periodontal disease in 34%, oral candidiasis in 24%, tooth loss in 24%, oral mucosal ulcers in 22%, taste impairment in 20%, xerostomia and salivary gland hypofunction in 14%, dental caries in 24%, and burning mouth sensation in 10% cases. Also, fasting and postprandial blood glucose levels were significantly higher in the group with oral manifestations than the group without oral manifestations. Furthermore, it was revealed that most patients with the oral manifestations of DM significantly developed neuropathy, retinopathy, and dyslipidemia.^6^ In the report, it was revealed that many patients with DM suffered oral manifestations, such as periodontitis and taste impairment, and ran the risk of developing other complications. Perros et al reported that electrical taste thresholds, detection threshold for glucose, and recognition threshold for glucose and salt were higher in newly diagnosed non–insulin‐dependent DM patients compared to that in the control subjects. After the treatment of hyperglycemia, median total HbA1 fell from 12.6% to 8.8% and subsequently impaired and improved each threshold. The authors concluded that this taste abnormality may influence the premorbid choice of nutrients, with a preference for sweet‐tasting foods, thereby exacerbating hyperglycemia.^7^ From these two reports, we believe that it is important to assess taste alteration for patients with diabetes.

Therefore, we considered that diabetes treatment also improved dysgeusia, and tongue condition (candidiasis and structural change of tongue surface) and that those conditions might be an index of improvement. We did not prescribe zinc nor iron and neither any antifungal drug but all the symptoms were improved by the diabetes treatment, the insulin injection, the dietary counseling, and therapeutic exercise. She succeeded in the intake of balanced diets and in exercising routinely, and it caused a reduction of 14 kg of weight. In dentistry, we performed oral care to reduce bacterial infection, bacterial examination, and record tongue condition routinely. We provided these results to the multidisciplinary team, such as physicians, nurses, nutritionists, dentists, and dental hygienists. We confirmed that her dysgeusia was recovered as well as the diabetes without any additional drug administration. All the multidisciplinary team could improve not only her disease but also her lifestyle in order to have a healthy life.

In conclusion, dysgeusia was improved by diabetes treatment and sharing oral examination information within the multidisciplinary team was useful for an adequate treatment. The assessment of oral conditions, including taste alteration, is useful for the treatment of patients with diabetes.

## CONFLICT OF INTEREST

The authors state that they have no conflict of interest.

## AUTHOR CONTRIBUTION

KM: was attending physician and performed bacterial examination analysis. YY: performed oral hygiene guidance and oral care. MT: was attending physician and made critical revision. KY: drafted the manuscript. IM: performed salt‐impregnated test and nutrition guidance. KI: made critical revision. SN: was attending physician and made critical revision. EM: performed oral hygiene guidance and oral care. KN: performed oral hygiene guidance and oral care. TK: made critical revisions and is the corresponding author. ST: made critical revisions and is the corresponding author.

## ETHICAL APPROVAL

This article does not describe any studies involving human participants or animals performed by any of the authors.
